# Serum Biomarkers Identification by Mass Spectrometry in High-Mortality Tumors

**DOI:** 10.1155/2013/125858

**Published:** 2013-01-15

**Authors:** Alessandra Tessitore, Agata Gaggiano, Germana Cicciarelli, Daniela Verzella, Daria Capece, Mariafausta Fischietti, Francesca Zazzeroni, Edoardo Alesse

**Affiliations:** Department of Biotechnological and Applied Clinical Sciences, University of L'Aquila, Via Vetoio Coppito 2, 67100 L'Aquila, Italy

## Abstract

Cancer affects millions of people worldwide. Tumor mortality is substantially due to diagnosis at stages that are too late for therapies to be effective. Advances in screening methods have improved the early diagnosis, prognosis, and survival for some cancers. Several validated biomarkers are currently used to diagnose and monitor the progression of cancer, but none of them shows adequate specificity, sensitivity, and predictive value for population screening. So, there is an urgent need to isolate novel sensitive, specific biomarkers to detect the disease early and improve prognosis, especially in high-mortality tumors. Proteomic techniques are powerful tools to help in diagnosis and monitoring of treatment and progression of the disease. During the last decade, mass spectrometry has assumed a key role in most of the proteomic analyses that are focused on identifying cancer biomarkers in human serum, making it possible to identify and characterize at the molecular level many proteins or peptides differentially expressed. In this paper we summarize the results of mass spectrometry serum profiling and biomarker identification in high mortality tumors, such as ovarian, liver, lung, and pancreatic cancer.

## 1. Introduction

Cancer-related mortality is one of the leading causes of death worldwide. The most effective treatment to fight cancer is still early diagnosis. On the other hand, it is known that the correct classification of the tumor, coupled to a suitable therapy and to a stringent follow-up, helps to prevent and detect relapses. Cancer is a very heterogeneous disease, and, at the diagnostic level, is defined by many indexes such as histological grade, tumor stage, patient age, sex and, more importantly, genetic background and profiles. Histological evaluation of tumor specimens obtained from tissue biopsy is the gold standard of diagnosis, but often tumors with the same histopathological features respond differently to the same therapy. New generation diagnostic platforms, previously unavailable, have enabled to better characterize transcriptomic signatures that predict tumor behaviour, helping to define diagnosis, prognosis, and the most appropriate therapies [1–3]. Tumor biomarker discovery in biological fluids, such as serum, plasma, and urine, is one of the most challenging aspects of proteomic research [4]. Many researchers have attempted to identify biomarkers in serum that reflect a particular pathophysiological state. Since the expressed proteins, native, fragmented, or posttranslationally modified, quickly change in response to environmental or pathological stimuli, the serum proteome is considered dynamic, oppositely to the stable nature of the genome. Proteins and their functions can determine the phenotypic diversity that arises from a set of common genes. The study of the serum proteome highlights differences in protein expression reflecting a specific pathological state and provides useful information to diagnose a disease, to evaluate prognosis or therapy response [5].

## 2. Biomarker Discovery in Cancer: The Complexity of Human Serum

Single or a small number of serum-based biomarkers indicative of cancer progression, such as prostate-specific antigen (PSA), alpha-fetoprotein (AFP), CA-125, CA-15.3, CA-19-9, or CEA for prostate, liver, ovary, breast, pancreas, or colon cancer, are currently used. Most of these molecules have been isolated from animals immunized with tumor cells extracts or cell lines, with subsequent screening for monoclonal antibodies against cancer-associated antigens [6]. The above-mentioned proteins increase the accuracy of diagnosis, even though there is an urgent need to isolate and use in clinical practice more specific biomarkers, or groups of biomarkers, to precisely characterize the disease at the diagnostic or prognostic level and to monitor its progression [7, 8]. Biomarkers could also help to predict the response of the patient to anticancer therapy and thereby to guide physicians in choosing the best treatment. This research is more appealing due to the simplicity of obtaining blood samples, but, at the same time, shows limits due to the complexity of the serum protein mixtures. A plethora of molecules from almost every tissue of the body can be found in human serum/plasma. Many of the serum proteins are present at very low concentrations (less than pg/mL), while others are present in very large amounts (more than mg/mL). Serum and plasma are very complex mixtures of proteins and exhibit a broad dynamic range of relative abundance (up to 12 orders of magnitude) [9]. They contain thousands of proteins, whose some are very abundant (e.g., albumin, immunoglobulins, apolipoproteins) and constitute approximately the 95% of the total protein content, but only the 0.1% of total protein species [10, 11]. For these reasons, it is thought that many potentially important proteins and markers, if present at low concentrations, can escape the detection. Evidences show that the circulating fragments from unmodified or post-translationally modified proteins generated in the tumor tissue microenvironment can be used as diagnostic or prognostic markers. Proteolysis within the tissue or deregulated post-translational events (e.g. phosphorylation) generate protein fragments that diffuse into the circulation and could give information about the presence or the progression of the disease, then facilitating the management of the tumor. Among these fragments, the fraction with low molecular weight, the peptidome (<20 KDa, LMW peptides), is protected from renal clearance by interaction with abundant serum proteins and, in particular, seems to be an important source of biomarkers [12].

In order to simplify the biomarker discovery process, many prefractionation methods have been developed to remove highly abundant proteins. These useful precursors of proteomic analysis include the use of immobilized dyes (cibacron blue) [13, 14], immunoaffinity-based techniques [15, 16], solid phase fractionation [17], liquid chromatography [18], or low-molecular weight fraction enrichment [19–21]. A promising method under investigation is based on the use of N-isopropylacrylamide (NIPA) nanoparticles which allow a fast one-step capture and concentration of analytes less than 20–25 KDa in molecular weight [22–24]. At the same time, the nanoparticles are able to protect the proteins from the degradation due to the “*ex vivo*” enzymatic activity of serum proteases, and, when conjugated with suitable chemical baits, show higher capability to sequester and retain many different proteins from whole serum, on the basis of their chemical and physical properties [25]. With this method, the captured analytes can be recovered by a simple electroelution and then analyzed by HPLC-MS/MS, western blotting or immunoassays for complete molecular characterization. To reduce the complexity of serum proteome, several studies are focused on targeting a specific subset of serum proteins [26]. Glycosylation is one of the most common posttranslational modifications in proteins. Approximately 50% of known eukaryotic proteins are glycosylated [27]. It is known that cancer cells express aberrant glycosylation patterns [28]. The analysis of glycosylated proteins (glycoproteome) has received great interest, because of the glycoproteic nature of the currently used cancer biomarkers. Two major methods have been developed to enrich glycoproteins or glycopeptides, based on chemical capture (reaction between aldehyde groups and hydrazide) [29, 30] and lectin-affinity capture (specific recognition of protein glycan moieties by lectins) [31].

## 3. Mass Spectrometry: The Cancer Biomarker Discovery Tool

Many studies have been focused on the identification of new serum biomarkers by mass spectrometry (MS). This powerful method enables to identify a protein without requiring the knowledge of its amino acid sequence. Further improvement of this technology has provided high accuracy to define mass-to-charge ratio (*m*/*z*) and to generate high-resolution spectra. In addition, the development of tandem mass spectrometry (MS/MS), able to provide *de novo *protein sequence information, has enhanced the applications of this technology in proteomics [32, 33]. Several MS methods have been used to characterize body fluids. Different combinations of ionization sources (e.g., MALDI, ESI), analysers (e.g., time of flight TOF, quadrupole, Fourier transform and quadrupole ion traps), and fragmentation methods (e.g., CID collision induced dissociation, ETD electron transfer dissociation) can be used. MALDI-TOF (Matrix-Assisted Laser Desorption/Ionization-time of flight) [34] is based on a soft ionization method where a laser beam generates evaporation of a crystallized sample-matrix mixture. MALDI is used in biochemical areas for the analysis of proteins, peptides and oligonucleotides. Surface-Enhanced Laser Desorption/Ionization Time-of-Flight (SELDI-TOF), a modification of MALDI-TOF, allows the identification of proteins differentially expressed in serum by applying a small amount of sample directly on an array surface involving various chromatographic models based on classical chemistries (i.e., normal phase, hydrophobic, cation- and anion-exchange), affinity-coated surfaces (IMAC, immobilized metal affinity capture), or biomolecular affinity probes [5, 35, 36], with minimal requirements for purification and separation [37]. Selectively retained proteins are analyzed by laser desorption and subsequent ionization. The results are shown by a mass spectrum identifying *m*/*z* ratios and peak intensities of peptides/proteins. Data preprocessing (i.e., calibration, baseline correction, normalization, peak detection, and alignment), bioinformatic and statistical analyses are performed in order to highlight and characterize any protein differentially expressed. SELDI-TOF allows the detection of many low molecular weight proteins [38]. Liquid chromatography/electrospray ionization tandem mass spectrometry (HPLC/ESI-MS/MS) technology is an alternative approach for serum biomarker identification. The mixtures of analytes are subjected to HPLC then the solution is nebulized under atmospheric pressure and exposed to a high electrical field which generates a charge on the droplets' surface [39]. Due to the evaporation, droplets become much smaller and enter into the analyzer. HPLC-ESI-MS/MS couples protein fractionation with mass spectrometry, where peptide sequence tags can be produced from peptide fragments. Tandem mass spectrometry and data analysis by suitable bioinformatic tools, algorithms and databases, provides a powerful method to characterize peptides at the aminoacidic level, allowing a highly refined analysis [40].

The use of mass spectrometry for serum biomarker discovery is quite simple: spectral peaks (plots representing on the *x*-axis the *m*/*z* ratios of ions, and on the *y*-axis the detected ion abundance), are identified in a pathological group and compared with those obtained from normal control groups. Differences in spectral profiles detect putative biomarkers. Essentially four strategies have been used in MS biomarker discovery: analysis of polypeptides separated by electrophoresis or chromatography, with or without prior fragmentation; analysis of enzymatic peptide fragments separated by HPLC and then analyzed by ESI or MALDI; analysis of proteins adsorbed on a solid surface; and analysis of specific serum fractions, such as the peptidome or the glycoproteic fraction [4].

Due to the lack of effective screening test, these methods have been applied to the serum biomarker discovery in many tumors, including those with high mortality, such as ovary, lung, liver, and pancreatic cancer. Here we report the results of studies focused on serum biomarker identification in the above-mentioned types of cancer. Several protein profiles and specific proteins (Tables 1, 2, 3, and 4) have been characterized and classified as putative biomarkers.

### 3.1. Serum Biomarkers in Ovarian Cancer

Despite advances in cancer therapy, mortality due to ovarian cancer is almost unmodified during the last decades [50]. Ovarian cancer is usually diagnosed at a late clinical stage in more than 80% of patients [51]. In this group, the 5-year survival is approximately 35%. By contrast, the 5-year survival for patients with stage I ovarian cancer is more than 90% and surgery alone can be used as elective therapy [52]. Cancer antigen 125 (CA-125) is the most widely used biomarker for ovarian cancer. Elevated levels of CA-125 are detected in about 80% of patients with advanced-stage disease, but they are increased in only 50–60% of patients with early stage ovarian cancer [53]. The calculated positive predictive value for CA-125, considered as a single marker, is less than 10%, but this value was improved by ultrasound screening methods [54].

#### 3.1.1. Proteomic Patterns for Ovarian Cancer Detection

Analysis of sera by TOF-MS provided specific signature patterns which can be compared to distinguish ovarian cancer from benign disease or normal individuals. A training set of 50 sera from unaffected women and 50 from patients with ovarian cancer were analyzed by SELDI/TOF by using a C16 hydrophobic interaction protein chip. An algorithm identified a proteomic pattern able to distinguish cancer from non-cancer subjects. The pattern was then used to differentiate an independent set of 116 samples (50 ovarian cancer, 66 non-malignant disease or unaffected), yielding 100% sensitivity and 95% specificity [67]. Some of the characteristic ion peaks in the previous signature have been sequenced and described in an independent cohort of ovarian cancer sera [36]. One hundred nine serum samples from ovarian cancer patients, 19 sera from individuals with benign tumors, and 56 from healthy donors were analyzed on strong anion-exchange surface using SELDI-TOF. Three panels of candidate protein biomarkers were obtained (1st: 4.4 kDa, 15.9 kDa, 18.9 kDa, 23 kDa, 30.1 kDa—95.7% sensitivity, 82.6% specificity; 2nd: 3.1 kDa, 13.9 kDa, 21.0 kDa, 79.0 kDa, and 106.7 kDa—81.5% sensitivity, 94.9% specificity; 3rd: 5.1 kDa, 16.9 kDa, 28 kDa, 93 kDa—72.8% sensitivity, 94.9% specificity). The protein panels correctly diagnosed 41/44 blind test samples: 21/22 malignant ovarian cancers, 6/6 low malignant potential tumors, 5/6 benign tumors, 9/10 normal individuals [68]. A four-peak model (*m*/*z* 6195, 6311, 6366, 11498), performing better than CA-125, has been identified for diagnosis or monitoring of the therapy in ovarian cancer. This study was conducted on a training (31 primary cancer, 16 benign ovarian disease, 25 healthy controls) and a blind test set (23 ovarian cancer, 15 benign, 5 normal). The four peak model showed a sensitivity of 90.8% and a specificity of 93.5% in the training set, and a sensitivity of 87% and a specificity of 95% in the blind test set in discriminating cancer from non cancer patients [69]. Hocker et al. [70] analyzed mass spectrometry peak differences in 35 ovarian cancer patients and 16 disease-free subjects. Proteomic profiles distinguished early-stage from advanced cancer with a sensitivity of 80% and a specificity of 93%.

#### 3.1.2. Glycoproteomics in Ovarian Cancer

An et al. [80] developed a glycomic approach to identify oligosaccharide markers for ovarian cancer by analyzing ovary cell lines supernatants and then confirming their presence in sera from patients and healthy controls. Changes in glycosilation were monitored by MALDI-Fourier Transform Ion Cyclotron Resonance-MS. Approximately 15 unique serum glycan markers were detected in all patients and were absent in controls. Leiserowitz et al. [81] analyzed glycan markers and CA-125 levels in 48 sera from ovarian cancer women and 24 controls. Oligosaccharides were cleaved from serum glycoproteins and isolated using solid phase extraction. MALDI-Fourier Transformation-MS was used to identify peaks. Sixteen unique oligosaccharide signals were identified in most of the cancer patients (44/48) and just in 1/24 controls (sensitivity 91.6%, specificity 95.8%).

#### 3.1.3. Ovarian Cancer Biomarker Proteins Involved in Inflammatory Processes

Several proteins involved in inflammatory processes and acute-phase response have been identified as putative biomarkers for ovarian cancer. In a study by Zhang et al. [36], sera were fractionated by anion exchange chromatography. Aliquots were bound in triplicate with a randomized chip/spot allocation scheme to IMAC3-Cu, SAX2, H50, and WCX2 protein chip array. For biomarker identification, proteins were purified, separated by SDS-PAGE, and analyzed by MS/MS. Three proteins/protein fragments, described as acute-phase reactants (apolipoprotein A1, *m*/*z* 28043, a truncated form of transthyretin, *m*/*z* 12828, and a fragment of inter-*α*-trypsin inhibitor heavy chain H4, *m*/*z* 3272), were identified as putative biomarkers able to improve the detection of early stage ovarian cancer [36]. Ye et al. [42] identified by SELDI an 11.7 kDa peak showing higher intensity in cancer sera. After protein purification and LC-MS/MS analysis, the alpha chain of haptoglobin, an acute phase reactant, was identified and further validated by western blot and ELISA as a potential biomarker for ovarian cancer, by using a specific polyclonal antibody against the peptide identified by MS/MS analysis. The marker was 2-fold-expressed in cancer sera and had 64% sensitivity and 90% specificity when used alone, or 91% and 95% sensitivity and specificity, if combined with CA-125. After high abundance protein removal and two-dimensional gel electrophoresis (2-DE), Ahmed et al. [43] performed nanoelectrospray quadrupole-quadrupole time of flight mass spectrometry (n-ESIQ-(q)TOF-MS) and MALDI/TOF analysis on six protein spots over-expressed in cancer sera. Protein isoforms of haptoglobin-I precursor (HAPI), a liver glycoprotein present in human serum, were identified as putative novel biomarkers and confirmed by 2-DE and western blotting on the serum from healthy controls and grade 1 and 3 ovarian cancer patients. Bergen III et al. [44] analyzed by nano-LC-ESI-TOF-MS or Fourier Tranform Ion Cyclotron Resonance (FT-ICR) the low molecular weight serum fraction obtained by ultrafiltration and identified several candidate biomarkers. Among these, the fibrinopeptide-A, already described as involved in acute phase reactions and elevated in many cancer including ovary, was detected. Two peaks (11.7 and 11.5 kDa) were identified by SELDI-TOF in thermostable plasma fractions from 27 ovarian cancer and 34 control sera. A method involving cysteine modifications, 2-DE, and HPLC allowed to characterize the peaks corresponding to serum amyloid A1, an acute phase reactant, and its N-terminal arginine truncated form [45]. Kozak et al. [41] identified by micro-LC-MS/MS four biomarkers for early stage ovarian cancer (transthyretin, apolipoprotein A1, transferrin, and beta-hemoglobin), corresponding to already described 13.9-TTR-, 12.9-TTR-, 15.9-Hb-, 28-ApoAI-, 79-Tf-kDa SELDI peaks [68]. Differential expression of these proteins in sera was also confirmed by western blot and ELISA. Lopez et al. [47] set-up a workflow using carrier protein-bound affinity enrichment of serum samples directly coupled with MALDI/TOF. Subsequent tandem MS analysis defined the serum protein-bound peptides' sequence. The procedure was able to identify several specific biomarker panels to differentiate stage I ovarian cancer from unaffected and age-matched women. Among the peptides identified, proteins involved in inflammatory processes (complement component 3 precursor, complement component 4A preprotein, inter-alpha globulin inhibitor H4), as well as the glycosyltransferase-like 1B, a peroxisomal oxidation enzyme (D-amino-acid oxidase), two proteins involved in cancerogenesis (transgelin 2, casein kinase II alpha 1 subunit isoform a), fibrinogen alpha chain isoform alpha preprotein, and keratin 2a were described as putative biomarkers. In a study on sera from patients with ovarian cancer, compared with sera from patients with benign masses or different type of cancer, it has been shown that the chemokines CC2 motif ligand 18 (CCL18) and CXC motif ligand 1 (CXCL1), identified by MALDI-MS/MS analysis and further validated by ELISA, can be considered as novel circulating tumor markers for differential diagnosis between ovarian cancer and benign masses (sensitivity 92%, specificity 97%) [48]. A nested case-control study performed on 295 sera from women pre-dating their ovarian cancer diagnosis and 585 matched control samples, showed that two peaks identified by MALDI, described as the connective tissue-activating peptide III (CTAPIII) and the platelet factor 4 (PF4), can be associated with CA-125 to improve early diagnosis [49].

#### 3.1.4. Ovarian Cancer Biomarker Proteins Involved in Other Functions

Woong-Shick et al. [46] studied by SELDI-TOF 35 sera from ovarian cancer patients in comparison to 10 from normal women. After protein purification and N-terminal sequencing, hemoglobin-*α* and *β* chains were described as corresponding to the most distinctive peaks differentially expressed (15.1 and 15.8 kDa). ELISA validation test for intact hemoglobin indicated a sensitivity of 77% in sera from ovarian cancer patients. As above-mentioned, hemoglobin was also described as a putative ovarian cancer biomarker in a study by Kozak et al. [41].

### 3.2. Serum Biomarkers in Liver Cancer

Liver cancer is often diagnosed at very late stage and is associated to poor prognosis, high recurrence and mortality. Hepatocellular carcinoma (HCC), the most frequent liver neoplasm, is the fifth most common cancer, affecting approximately one million people every year, with an incidence almost corresponding to death rate [94] and a 5 year survival ranging from 17% to 50%. Some predisposing factors, such as viral infections [95], diabetes, metabolic syndromes, exposition to aflatoxin, or alcohol consumption, are frequently related to liver tumor initiation. This allows a management of the patients at risk, making it possible in some cases to diagnose the disease earlier. The most important liver cancer serum biomarker is alpha-fetoprotein (AFP), an oncofetal glycoprotein with elevated levels in patients affected by cirrhosis and HCC. The sensitivity and specificity of AFP range between 60–80% and 70–90%, respectively [96]. For this reason, AFP test utility for screening procedures is questionable. Many studies have been focused on characterizing differentially expressed proteins in sera from patients affected by liver cancer or predisposing diseases and, similarly to ovarian cancer, proteomic profiles and proteins have been identified.

#### 3.2.1. Proteomic Patterns for Liver Cancer Detection

A total number of 117 HCV-positive sera from 39 patients affected by low-grade fibrosis, 44 with cirrhosis without HCC, and 34 with both cirrhosis and HCC were preprocessed by anion-exchange fractionation and analyzed by SELDI-TOF. A four markers panel (7486, 12843, 44293, 53598 Da) identified HCC with a sensitivity of 100% and a specificity of 85% in a two-way comparison of HCV-cirrhosis versus HCV-HCC training set. Sensitivity and specificity for the correct identification of HCC were 68% and 80% for random test samples. Fibrosis patients were distinguished from cirrhotic using a five marker panel (2873, 6646, 7775, 10525, 67867 Da, sensitivity and specificity 100% and 85%, 80% and 67% in the training and random test samples, resp.). After purification, MS/MS analysis and immunoassay validation, the 6646 Da protein was identified as apolipoprotein C-I and described as a marker to differentiate liver fibrosis from cirrhosis [97]. SELDI-TOF protein-chip technology was applied to analyze sera from patients affected by HCV-associated chronic liver diseases with (64 samples) or without (77 samples) HCC. Samples were randomly split into two analysis groups. Six selected protein peaks (*m*/*z* 3444, 3890, 4067, 4435, 4470, 7770) gave information to perform early diagnosis and to distinguish HCC from chronic liver disease in the absence of HCC (sensitivity and specificity 83% and 76%). The model was also applied to the analysis of sera from 5 subjects HCC-free and from 7 HCC patients collected before the diagnosis by ultrasonography. The markers allowed to correctly predict the presence of HCC in 6/7 patients [98]. Wu et al. [99] identified serum proteins and peptide profiles to differentiate HBV-related HCC and HBV-related cirrhosis. Forty-five protein peaks distinguished HCC from LC (liver cirrhosis) samples. The most significant SELDI 3892 *m*/*z* peak showed sensitivity and specificity of 69% and 83% and was identified also in six AFP-negative patients. The 3892 peak was considered as a complementary diagnostic marker or a potential marker for positive or negative *α*-fetoprotein HCC. SELDI-TOF analysis of sera from 120 patients affected by HCC and 120 affected by cirrhosis showed five proteomic peaks (*m*/*z* 3324, 3994, 4665, 4795, and 5152) able to achieve, especially for early stage HCC, a diagnostic value better than serum AFP (83% sensitivity and 92% specificity in the test set) [100]. Cui et al. [101] formulated classification trees, based on SELDI serum protein profiles, able to distinguish patients affected by chronic hepatitis B, cirrhosis, and HCC from healthy individuals. Samples were divided into training and testing groups, each composed by HBV, liver cirrhosis, HCC patients matched with normal controls. Decision trees distinguished HCC with 90% sensitivity and 89% specificity, cirrhosis with 100% sensitivity and 86% specificity, and HBV patients with 85% sensitivity and 84% specificity.

#### 3.2.2. Glycoproteomics in Liver Cancer

Goldman et al. [102] used a glycomic approach to evaluate the abundance of 83 N-glycans in a total of 202 sera from 73 HCC patients, 52 with chronic liver disease and 77 controls. Glycans were enzymatically obtained from serum and permethylated before MALDI-TOF analysis. The abundance of 57 N-glycans resulted significantly altered in HCC samples. Six glycans were used to differentiate HCC cases from controls and showed sensitivity and specificity of 73–90% and 36–91%, respectively. A combination of three N-glycans (*m*/*z* 2472.9, 3241.9, 4052.2) was able to classify HCC with 90% and 89% sensitivity and specificity in an independent validation set of patients with chronic liver disease. Two-hundred and three serum samples collected from 73 HCC cases, 52 chronic liver disease, and 78 healthy subjects were treated for N-glycans releasing and then analyzed by MALDI. Seven glycan peaks achieved good performance in distinguishing HCC from chronic liver disease patients and normal individuals [103]. After depletion of high abundance proteins, Liu et al. [55] analyzed 27 sera from early HCC in comparison to 27 cirrhosis patients in order to identify glycoprotein biomarkers. A lectin array of 16 selected lectins was used to define glycan structures showing changes between the two groups of samples. Samples were then analyzed by exactag labeling, lectin extraction and LC-MS/MS. Complement C3, ceruloplasmin, histidine rich glycoprotein (HRG), CD14 and hepatocyte growth factor (HGF), as validated by western blot, were considered putative biomarkers in differentiating early HCC from cirrhosis with a sensitivity of 72% and a specificity of 79%.

#### 3.2.3. Liver Cancer Biomarker Proteins Involved in Inflammatory Processes

Paradis et al. [56] studied by SELDI-TOF eighty-two sera from patients with cirrhosis, either without (38 samples) or with (44 samples) HCC. Thirty protein peaks significantly differentiated cirrhotic patients affected by HCC from those unaffected. An algorithm showing the six highest scoring peaks allowed the correct classification of patients with or without HCC in 92% of individuals in the test set and in 90% in the validation set. After sera fractionation (IMAC-Zn spin column), analysis on NP20 chip array and protein recovery from tricine SDS-PAGE, tandem MS was performed and the highest discriminating peak (8.9 kDa) was described as the C-terminal part of the V10 fragment of vitronectin, a protein involved in cell adhesion, humoral defense mechanism as well as cell invasion. SELDI-TOF was used to identify differentially expressed proteins in hepatocarcinoma (55 samples in total, 31 HBV-related and 24 HCV-related) and chronic hepatitis patients (18 HBV and 30 HCV). After serum fractionation by anionic exchange chromatography, the proteins were characterized by 2-DE separation and LC-MS/MS analysis. The protein complement C3a (about 8.9 kDa), elevated both in chronic HCV and HCV-related HCC patients, was identified as a candidate biomarker and further validated by PS20 chip immunoassay and western blot [57]. Yang et al. [58] used 2-DE combined to nano-HPLC-ESI-MS/MS to identify 14 proteins differentially expressed (12 up and 2 down-regulated) in HCC patients with respect to normal controls. On the other hand, using whole serum trypsin-digested and then analyzed with nano-HPLC-ESI-MS/MS, twenty-nine proteins were identified with high levels of confidence. Six of them (Annexin VI isoform, Complement component 9, Ceruloplasmin-ferroxidase-, Serum amyloid A4, Serum amyloid A2, Serum amyloid A1 isoform 2), playing a role in immune and acute phase response or in membrane dynamics along endocytosis or exocytosis pathways (Annexin VI), were detected only in HCC patients. An 11 peak algorithm, generated by SELDI/TOF analysis, distinguished patients with HCC (41 samples) from those with hepatitis C cirrhosis (51 samples) better than the currently used biomarkers AFP, AFP L3 (Lens culinaris agglutinin-reactive AFP) and PIVKA-II (prothrombin induced by vitamin K absence-II). Within the 11-protein signature, the 13.4 kDa feature was purified, identified as cystatin C by MS/MS analysis and further validated by ELISA. The cystatin C, a cysteine protease inhibitor marker of inflammation as well as renal function, resulted overexpressed in HCC samples and was described as a marker to distinguish HCC from HCV-related cirrhosis patients [59]. He et al. [60] performed by SELDI-TOF serum profiling on 81 patients with HBV-related HCC and 33 normal controls, randomly split into a training and a testing set. Six proteomic peaks (*m*/*z* 3157.33, 4177.02, 4284.79, 4300.80, 7789.87, 7984.14) were considered to construct the best classification tree (sensitivity 95%, specificity 100% in the testing set). Protein fraction corresponding to the 7489 *m*/*z* peak was isolated and characterized by MS/MS analysis as the inflammatory cytokine neutrophil activating peptide 2 (NAP-2). NAP-2 was validated by immunohistochemistry in HCC tissues and resulted specifically associated to hepatitis B-related HCC [60]. Sera from eighty-one patients with HBV-related HCC and 80 healthy controls were divided in two sets and analyzed by SELDI-TOF. Candidate biomarkers were purified and identified by MS/MS and database searching. Two proteins, the thrombin light chain (*m*/*z* 4096) and the chemokine growth-related oncogene alpha (GRO-alpha) (*m*/*z* 7860) were selected as putative biomarkers. A clinical validation set composed by 48 HCC, 54 liver cirrhosis, 151 patients with other cancers and 42 healthy donors was analyzed to confirm data by SELDI-immunoassay. The proteins, when associated to AFP, resulted in a sensitivity of 91.7% and a specificity of 92.7% [61]. Sera from cirrhosis and HCC patients were analyzed by cleavable stable isotope labeling (cICAT) coupled to LC-ESI-MS/MS. Among 31 proteins differentially expressed, the alpha-1 acid glycoprotein (AGP), an acute phase reactant, was chosen for western blot assay and validation in a separate study. AGP was useful for discrimination of HCC from cirrhosis in patients with AFP less than 500 ng/mL [62]. A study based on 2D-gel electrophoresis and MALDI-TOF in patients with hepatocarcinoma or liver cirrhosis revealed five proteins differentially expressed (haptoglobin, Hp2, preprohaptoglobin, SP40 and SAA1). Western blot analysis showed haptoglobin, the most representative protein, as overexpressed in HCC patients. When used in association to AFP, the molecule improved the diagnostic accuracy. Serum haptoglobin also showed diagnostic potential in AFP-negative patients [63]. Several peptides in the serum low molecular weight fraction were identified by MALDI and then characterized by LC-MS/MS. Differentially expressed peptides were described as truncations of N terminus of complement C3f, a fibrinopeptide, complement C4alpha peptides, a zyxin peptide, a coagulation factor XIII peptide, and a biliverdin diglucuronide [64]. 

#### 3.2.4. Liver Cancer Biomarker Proteins Involved in Other Functions

Feng et al. [65] used a strategy based on sonication, albumin and immunoglobulin depletion, 2-DE and MALDI-TOF MS/MS to analyze 20 sera, respectively, from HCC, hepatitis B (HBV) patients and normal subjects. The same number of additional sera from corresponding groups was used for the validation test. Height proteins, involved in inflammatory processes or classified as acute phase reactants (alpha-1 antitrypsin, clusterin, ceruloplasmin, haptoglobin alpha2 chain, transferrin, and transthyretin) as well as alfa-fetoprotein and the heat-shock protein 27, a stress-inducible protein acting in thermotolerance, cell proliferation, and apoptosis, were differentially expressed in the above-mentioned groups. Validation by western blot analysis revealed HSP27 expressed in 90% of HCC, in 10% of HBV and in none of normal sera. Wu et al. [66] compared by 2-DE and mass spectrometry sera from HCC patients and normal controls. Eight protein spots differentially expressed were analyzed and four proteins were identified as putative biomarkers (MYH2 protein, mitochondrial ATP synthase, sulphated glycoprotein-2-clusterin SGP-2-, and glial fibrillary acidic protein (GFAP). SGP-2, known to be involved in inflammation and in the regulation of cellular proliferation, was also confirmed by immunoblotting in an independent set of samples.

### 3.3. Serum Biomarkers in Lung Cancer

Lung cancer is one of the leading causes of cancer-related mortality worldwide and is responsible for 1.3 million deaths worldwide annually [104, 105]. The poor prognosis is evidenced by the 5-year survival rate which is less than 15% [106]. Lung cancers are grouped into small-cell lung cancer (SCLC) and non-small-cell lung cancer (NSCLC), consisting of adenocarcinomas, squamous cell carcinomas, and large-cell carcinomas [107, 108]. NSCLCs comprise approximately 80% of all lung cancers [50], with adenocarcinomas and squamous cell lung cancers each accounting for approximately 30%. Many serologic biomarkers of lung cancer have emerged recently: these include carcinoembryonic antigen (CEA), the cytokeratin 19 fragment CYFRA21-1, cancer antigen CA-125 [109], plasma kallikrein [110], progastrin-releasing peptide (ProGRP), and neuron-specific enolase (NSE) [111].

#### 3.3.1. Proteomic Patterns for Lung Cancer Detection

In a study on 208 sera (158 lung cancer, 50 healthy controls), Yang et al. [112] identified a 5 proteins peak pattern (11493, 6429, 8245, 5335, 2538 Da) which, in a blind test, achieved sensitivity of 86.9% (79% for stage I/II lung cancers), specificity of 80% and positive predictive value of 92.4%. In particular, the pattern sensitivity was 91.4% in the detection of NSCLC. In a group of sera (54 SCLC, 24 NSCLC, 32 pneumonia patients, and 40 healthy subjects), SELDI-TOF spectra data analyzed by support vector machine (SVM) gave three patterns able to distinguish SCLC from pneumonia, NSCLC patients and from healthy individuals better than neuron specific enolase (NSE). The sensitivity and specificity ranged from 88% to 83% and from 91% to 75%, respectively [113]. A 17 MS protein signature was identified in a study on 139 lung cancer patients (stage III-IV), 158 healthy individuals and then validated in two sets of 126 (63 lung cancer stage III-IV, 63 controls) and 50 (25 lung cancer stage I-II, 25 controls) individuals. The signature distinguished lung cancer patients from normal subjects and showed sensitivity and specificity of 87.3% and 81.9% in the first validation set, and 90% and 67% in the second one [114]. Du et al. [115] captured and concentrated serum peptides by using magnetic beads-based weak cation exchange on the ClinProt robotic platform. The peptides were analyzed by MALDI-TOF. A 5 protein fingerprint distinguished SCLC patients (30 samples) from healthy individuals (44 samples) with a specificity of 97% and a sensitivity of 90%. In particular, 89% of stage I/II SCLC were correctly diagnosed.

#### 3.3.2. Glycoproteomics in Lung Cancer

Glycoproteomic approaches have been applied to identify biomarkers for NSCLC early diagnosis. After immunoaffinity depletion of highly abundant serum proteins, glycoproteins were captured and enriched by hydrazide chemistry, recovered and then analyzed by LC-MS/MS. Thirty-eight glycopeptides from 22 proteins distinguished cases from controls. Three of these proteins (alpha-1-antichymotrypsin ACT, insulin-like growth factor-binding protein 3 IGFBP3, lipocalin-type prostaglandin D synthase L-PGDS) were verified by ELISA, showing correlation with MS results [116]. 

#### 3.3.3. Lung Cancer Biomarker Proteins Involved in Inflammatory Processes

Howard et al. [71] identified by MALDI-TOF a peak (*m*/*z* 11702) differentially expressed in lung cancer patients (24 subjects) with respect to individuals with no evidence of cancer (17 subjects). After purification and peptide mapping, the peak was described as the acute phase reactant serum amyloid protein A and further validated by ELISA. Bharti et al. [77] analyzed by MALDI-TOF differentially expressed albumin-depleted serum proteins from SCLC patients and controls, recovered from a silver-stained SDS-PAGE. The peptides were characterized by sequencing. Haptoglobin *α*-subunit, validated by immunoblot, was considered as a biomarker with its level correlating with the disease stage. In addition, they analyzed by ELISA the levels of hepatocyte growth factor (HGF), a multifunctional protein which regulates both cell growth and motility, and described it as a potential SCLC biomarker. A study performed on 218 sera from 175 lung cancer patients and 43 controls by SELDI-TOF showed an 11.6 kDa protein peak significantly elevated in cancer sera and increased in association to the clinical stage. Serum amyloid A protein was identified by tricine SDS-PAGE and MALDI-MS/MS analysis as a biomarker to discriminate lung cancer patients from healthy individuals. The marker was validated by immunoprecipitation and ELISA in the same samples and showed sensitivity of 84% and specificity of 80% [72]. In a study on 227 sera (146 lung cancer, 41 benign lung disease, 40 normal subjects) three peaks differentially expressed (*m*/*z* 13780, 13900, 14070) were identified by SELDI. The peaks corresponded to native transthyretin (negative acute-phase reactant) and its two variant, as demonstrated by SDS-PAGE and ESI-MS/MS analysis and further validated by immunoprecipitation and immunoblotting. The transthyretin expression was significantly lower in lung cancer sera compared with sera from normal individuals, but higher compared with those obtained from benign lung disease. Subsequent ELISA assay indicated that the levels of transthyretin were consistent with those obtained by SELDI, showing approximately 65–75% sensitivity and specificity [78]. The diagnostic accuracy of MALDI in analyzing unfractionated serum was assessed in a study by Yildiz et al. [117] performed on 142 sera form lung cancer patients matched with 146 samples from normal controls. Samples were split into training and test set. A serum proteomic signature of seven features achieved an overall accuracy of 78% and 72.6% in the training and blinded test set, respectively. The peptides around 11500 Da were further analyzed by using SDS-PAGE separation and LC-MS/MS and described as a cluster of truncated forms of serum amyloid A protein. In a study on 154 sera from pre-treated patients (55% early, 45% advanced-stage) an isoform of serum amyloid A, corresponding to an 11.6 kDa SELDI peak and characterized by SDS-PAGE and tandem MS, was found to be elevated in patients with poor prognosis. In this study, sera were prefractionated in six protein fractions on the basis of their isoelectric points [73]. Forty-nine proteins were found to be differentially expressed by LC-ESI-MS/MS in pools of sera from nonsmall cell lung cancer (adenocarcinoma and squamous cell carcinoma) with respect to healthy controls. Multiple reaction monitoring (MRM) assay was used to confirm the abundance of four selected proteins (serum amyloid A—SSA-, alpha-1 acid glycoproteins 1 and 2—AAG 1 and 2-, clusterin—CLU-). SSA and AAG 1 and 2 showed higher spectral count in lung cancer serum pool [74]. Analysis by SELDI-TOF of 227 sera showed 5 peaks (*m*/*z* 11530, 11700, 13780, 13900, 14070) identifying native serum amyloid A protein and transthyretin, and some of their variants as lung cancer biomarkers [75]. Serum amyloid A1 and A2 proteins were identified by LC-ESI-MS/MS in lung cancer pooled sera after SDS-PAGE fractionation. The levels were higher in lung cancer patients with respect both to patients affected by other pulmonary diseases or different cancers and to healthy controls. The results were confirmed by ELISA. Moreover, SSA expression in lung cancer samples was detected by tissue-microarray analysis [76].

#### 3.3.4. Lung Cancer Biomarker Proteins Involved in Other Functions

Ueda et al. [79] described a method based on enrichment of the peptidomic fraction and analysis by nano-LC-MS/MS. After further characterization by MRM-based relative quantification, peptides from apolipoprotein A4 (APOA4), fibrinogen alpha chain (FIBA), and limbin (LBN), a positive regulator of the hedgehog signaling pathway, have been identified as useful biomarkers for early detection and staging of lung tumors. 

### 3.4. Serum Biomarkers in Pancreatic Cancer

Pancreatic cancer is the fourth leading cause of cancer death in both men and women. The high mortality associated with it can be essentially attributed to advanced stage of disease at patient presentation. Few patients with pancreatic cancer are cured without surgical resection. The overall 5-year survival is about 5%, and only 20% of patients are candidates for surgical resection and possible treatment. For this small percentage of patients undergoing resection, even when followed by multimodal therapy, 5-year survival rates are still less than 25% [118–120]. Current methods for diagnosing pancreatic cancer are relatively ineffective to identify small potentially curable lesions. The marker recommended in clinical practice is serum CA-19-9. However, this marker is of little utility in establishing early diagnosis [121]. To date, there are no efficient modalities to early detect pancreatic cancer and strategies to improve survival have focused just on chemotherapy in the neoadjuvant setting or after resection.

#### 3.4.1. Proteomic Patterns for Pancreatic Cancer Detection

In a study on 15 healthy controls, 24 cancer and 11 chronic pancreatitis patients prospectively collected, the low molecular serum fraction was enriched and analyzed by MALDI-TOF. An eight peaks serum signature (*m*/*z* 4470, 4792, 8668, 8704, 8838, 9194, 9713, 15958) differentiated cancer patients from normal individuals (sensitivity and specificity of 88% and 93%), cancer from pancreatitis patients (sensitivity and specificity of 88% and 30%), and cancer from healthy plus pancreatitis-affected individuals (sensitivity and specificity of 88% and 66%). The most significant peak, *m*/*z* 9713, was described by MS/MS analysis as the apolipoprotein CIII [82]. Liu et al. [122] used SELDI-TOF technology to differentiate cancer from different pancreatic conditions, by studying 118 serum samples, split in training and test set. Two MS patterns, differentiating pancreatic adenocarcinoma from healthy controls and chronic pancreatitis, yielded in the test set sensitivity and specificity of 91.6% (cancer versus controls) and sensitivity of 90.9%, specificity of 80% (cancer versus chronic pancreatitis).

#### 3.4.2. Pancreatic Cancer Biomarker Proteins Involved in Inflammatory Processes

An orthotopic nude mouse model of human pancreatic cancer was used to detect serum biomarkers [83]. Mice were injected with a human pancreatic cancer cell line and then were divided in groups treated with anti-cancer drugs for some weeks. Sera were recovered and analyzed by SELDI Proteinchip technology. Plasma from 135 pancreatic cancer patients and 113 healthy individuals were at the same time examined. An 11.7 kDa protein peak, correlating with tumor weight, was detected in mice sera. After purification and separation by SDS-PAGE, the corresponding protein was identified as serum amyloid protein A and confirmed by western blotting. The level of SAA detected in plasma of pancreatic cancer patients correlated with the clinical stage. Ninety-six sera from pancreatic cancer patients undergoing surgery were fractionated by chromatography and analyzed by SELDI in comparison with as many sera from healthy controls. Twenty-four differentially expressed peaks were identified. Twenty-one of them resulted in downregulated pancreatic cancer samples. After purification, several proteins were identified by peptide mapping and postsource decay-matrix-assisted laser desorption ionization-TOF-MS. Down-regulated apolipoprotein A-II, transthyretin, and apolipoprotein A-I were described as potential markers in pancreatic cancer [84]. Hanas et al. [85] studied sera from pancreatic cancer patients by gel electrophoresis in order to highlight protein bands differing quantitatively. The proteins were analyzed and characterized by ESI ion-trap tandem MS. Three high mass proteins (*α*-2 macroglobulin, ceruloplasmin, complement 3C) were elevated in cancer sera with respect to controls. The ESI-MS analysis revealed great heterogeneity especially in the low mass region. By statistical analysis, twenty low-mass serum peaks correlating to controls and 20 different peaks correlating to cancer sera were found. A study performed by Fiedler et al. [86] on forty sera from patients matched with forty samples from healthy controls was focused on MALDI-TOF peptidome profile analysis after using magnetic beads for protein fractionation. Data were validated by using an additional 20 plus 20 sera set. Two significant peaks (*m*/*z* 3884 and 5959) showed 86.3% sensitivity and 97.6% specificity for discriminating patients from controls. The 3884 peak was described and further validated by immunoassay as platelet factor 4 (PF4). PF4, used in combination with CA-19-9, significantly improved sensitivity and specificity for the identification of pancreatic cancer [86]. Rong et al. [87] used immunoaffinity depletion of highly abundant proteins and 2-DE to identify 16 protein spots differentially expressed (8 iper- and 8 ipoexpressed in cancer sera). The proteins were analyzed and sequenced. Mannose-binding lectin 2 and myosin light chain kinase 2, a serine/threonine kinase, were identified as potential biomarkers for the pancreatic cancer diagnosis and further validated by western blot in an independent set of sera from pancreatic cancer patients and normal controls. Low molecular weight (<60 kDa) serum proteome from a training set composed by 24 patients with pancreatic cancer and 21 controls was analyzed by HPLC-ESI-MS/MS. Among many peaks identified, a peptide from CXC chemokine ligand 7 (CXCL7) was significantly reduced in cancer sera. Data were confirmed by high-density protein microarray in a large cohort of 140 patients affected by pancreatic cancer, 10 patients with chronic pancreatitis and 87 healthy controls. Combination of CXCL7 and CA-19-9, improved the discriminatory power for pancreatic cancer [88]. In order to limit the complexity of the plasma proteome, Pan et al. [89] employed multidimensional fractionation followed by HPLC-MS/MS. Many proteins/peptides were identified with this method. A group of differentially expressed proteins was selected and evaluated on a separate cohort of samples from pancreatic cancer, chronic pancreatitis patients, and nonpancreatic disease control. A composite marker of the tissue inhibitor of metalloproteinases TIMP1 and the adhesion molecule ICAM1, as characterized by ELISA, showed significant better performance than CA-19-9 in distinguishing pancreatic cancer, pancreatitis, non-pancreatic diseases and healthy controls. Forty-five samples from patients affected by pancreatic cancer and 20 from healthy controls were analyzed by 2-DE and LC-MS/MS. Seven protein spots were differentially expressed. Serum isoforms of alpha-1-antitrypsin (AAT), also confirmed by western blot, were described as upregulated and potential serum biomarkers for pancreatic cancer [90]. 

#### 3.4.3. Pancreatic Cancer Protein Biomarkers Involved in Other Functions

Bloomston et al. [91] analyzed by high-resolution 2-DE thirty preoperative sera from pancreatic cancer and thirty-two from healthy individuals. Differentially expressed spots were recovered and analyzed by MALDI-TOF and LC-MS/MS. Approximately 150 proteins resulted commonly overexpressed in all cancer patients. Four proteins discriminated 100% of pancreatic cancer and 94% of normal samples. Among them, fibrinogen-*γ* was identified as putative biomarker and further validated by enzymatic analysis in sera and immunohistochemistry in tumor tissues. One hundred and twenty-six sera form pancreatic cancer patients (84 with diabetes) were examined by SELDI-TOF in comparison to 61 sera from chronic pancreatitis (32 with diabetes), 24 from type 2 diabetes mellitus patients, and 12 from healthy controls. Classification algorithms obtained by MS analysis resulted to improve the diagnostic accuracy of CA-19-9 in pancreatic cancer diagnosis and to facilitate the differential diagnosis between pancreatic cancer and type 2 diabetes mellitus. Among the large number of peptides, that described with the *m*/*z* 3519 was identified as a member of the EGF-like family [123]. Fifty-eight sera from patients with pancreatic cancer were compared with 18 samples from patients affected by benign disease and 51 healthy controls. Sera were analyzed using a strong anionic exchange chromatography protein-chip and SELDI-TOF. Sixty-one protein peaks were detected to construct multiple classification trees to distinguish the disease groups, reaching 83% sensitivity and almost 100% specificity in discriminating cancer from controls and benign disease. Putative protein biomarkers were identified: one (*m*/*z* 4016) showed a downregulated trend in preoperative versus post-operative sera, three (*m*/*z* 4155, 4791, 28068) were detected in the differential diagnosis of the 3 test groups. C14orf166, a protein involved in modulation of mRNA transcription by Polymerase II, was identified as corresponding to the 28068 peak by ProteinChip immunoassay. The molecule showed levels significantly higher in pancreatic cancer patients, as confirmed by immunoenzymatic methods. C14orf166 was also iper-expressed in tumor cells [92]. A SELDI-TOF protein panel derived from the study of a training set composed by 38 pancreatic cancer sera, 54 disease controls, and 68 healthy volunteers was further validated on a first validation set (40 pancreatic cancer, 21 disease controls, 19 healthy volunteers) and then, by ELISA, on a second one (33 pancreatic cancer, 28 disease controls, 18 healthy volunteers). Some proteins corresponding to peaks of interest were purified and identified. A simplified diagnostic panel comprising CA-19-9, apolipoprotein C-I, apolipoprotein A-II, additionally validated by ELISA on the second validation set, resulted to improve the diagnostic ability of CA-19-9 [124]. Potential prognostic markers were initially identified by nano-LC-MS/MS in 4 groups of sera, each from 10 patients, selected on the basis of survival (long or short) and therapy (gemcitabine plus bevacizumab, or gemcitabine plus placebo). Alpha1-antichymotrypsin (AACT) was negatively correlated with overall survival and considered as a prognostic marker for pancreatic cancer [93].

## 4. Conclusions

Cancer is one of the leading causes of death worldwide. Advances in screening methods significantly improved early diagnosis with consequent enhancement of prognosis, survival and treatment efficacy. Unfortunately, some tumors are difficult to diagnose before the disease is in advanced or metastasizing state. Therefore, there is an urgent need to discover novel biomarkers which provide sensitive and specific disease detection. Over the past decade, serum biomarkers have been identified in sera from cancer patients by using powerful high-throughput technologies. Mass spectrometry allowed the identification of hundreds of proteins within complex biological samples such as tissues, serum, plasma, and urine. MS analytical attributes in biomarker discovery are its high mass accuracy, resolution and ability to characterize the peptides at the level of their aminoacidic sequence. Several workflows including methods for serum samples preparation (e.g., high abundance protein removal, serum fractionation), SDS-PAGE and 2D-GE, LC, different MS platforms, and protein chip arrays have been used for biomarker discovery. Many differential MS peak profiles were identified and several proteins were characterized and described as potential biomarkers for high mortality tumors (Tables 1–4), achieving different levels of sensitivity and specificity to diagnose the disease. Many of these proteins are involved in fundamental processes such as inflammation, cellular differentiation and proliferation, and apoptosis. Among them, positive (i.e., serum amyloid A, ceruloplasmin, complement factors, haptoglobin) and negative acute-phase reactants (i.e., transthyretin, transferrin) were differentially expressed in sera from ovary, lung, liver, and pancreatic cancer patients. Some putative ovarian cancer biomarkers described in this paper, such as the keratin 2a, the glycosyltransferase-like 1B, involved in glycosylation processes, and the casein kinase alpha 1, a serine/threonine kinase involved in cellular differentiation, proliferation and apoptosis, have been associated with processes related to cancer [125–128]. Similarly, the mannose binding lectin 2 (MBL2), a mediator of inflammation which results iperexpressed in pancreatic cancer sera, is involved in cancer processes. Genetic alterations of MBL2 can increase colon cancer susceptibility in African Americans and a MBL genetic polymorphism, associated to a reduction of vaginal MBL concentration, may be a risk for development of ovarian cancer [129, 130]. The chemokine CCL18, here described as candidate ovarian cancer serum biomarker, was also considered as a urine biomarker for bladder cancer detection [131]. Likewise, the HCC biomarker cystatin C, an inhibitor of cystein proteinases, showed significantly higher levels also in sera from lung cancer patients [132], and the HCC and lung cancer putative biomarker alpha-1 acid glycoprotein 1, an acute phase protein, was found as well elevated in sera and tumor tissues from patients affected by gastric carcinoma [133]. The here described liver cancer biomarker heat shock protein 27, a protein with cytoprotective and anti-apoptotic activity, measured by immunoenzymatic assay, was confirmed to be elevated in an independent cohort of sera from HCC patients [134]. 

Despite the great advances in the application of MS in serum biomarker discovery, several challenges remain. The identification of differential serum protein profiles and specific molecules able to discriminate normal from diseased subjects requires a technology able to highlight small differences and to process large series of serum samples. Although MS is the most powerful approach for biomarker identification, there are some boundaries in the analysis of serum. These can be attributable to the complex nature of serum and its tremendous dynamic range, to diurnal variation in protein expression, instability of proteins due to *in vivo* or *ex vivo* protease activity, pre-analytical methods reproducibility as well as to the intrinsic MS sensitivity (>*μ*g/mL) [135] in detecting analytes which usually range between 50 pg/mL and 10 ng/mL [136]. Accurate selection of cases and controls, standardization of sample collection and storage conditions, utilization of adequate and effective methods focused on reducing the complexity of serum/plasma prior MS analysis, use of different protein array with complementary binding conditions, refined bioinformatic and statistical analysis to process data, and suitable validation workflows by immunoassay on larger sets of independent samples are necessary elements to circumvent criticisms and improve the biomarker discovery process.

## Figures and Tables

**Table 1:** ovarian cancer serum biomarkers identified after MS-based studies.

Putative ovarian cancer biomarker	Expression in ovarian ca sera	References
Apolipoprotein I	Decreased	[36, 41]
Transthyretin	Decreased	[36, 41]
Inter-*α*-trypsin inhibitor heavy chain H4 (cleavage fragment)	Increased	[36]
Haptoglobin-*α* chain	Increased	[42]
Haptoglobin I precursor	Increased	[43]
Fibrinopeptide A	Increased	[44]
Serum amyloid A1	Increased	[45]
Hemoglobin *α* and *β* chain	Increased	[41, 46]
Transferrin	Decreased	[41]
Keratin 2a	Increased	[47]
Glycosyltransferase-like 1B	Increased	[47]
Complement component 3 precursor	Decreased	[47]
Complement component 4A preprotein	Decreased	[47]
Casein kinase II alpha 1 subunit isoform a	Decreased	[47]
D-amino-acid oxidase	Decreased	[47]
Transgelin 2	Increased	[47]
Inter-alpha (globulin) inhibitor H4	Increased	[47]
Fibrinogen, alpha chain isoform alpha preprotein	Increased	[47]
CC2 motif ligand 18 (CCL18)	Increased	[48]
CXC motif ligand 1 (CXCL1)	Increased	[48]
Connective tissue-activating peptide III (CTAPIII)	Decreased	[49]
Platelet factor 4 (PF4)	Decreased	[49]

**Table 2:** Liver cancer serum biomarkers identified after MS-based studies.

Putative liver cancer biomarker	Expression in liver ca sera	References
Complement c3	Increased	[55]
Histidine rich glycoprotein	Increased	[55]
CD14	Increased	[55]
Hepatocyte growth factor	Increased	[55]
C-terminal part of vitronectin V10 fragment	Increased	[56]
Protein complement C3a	Increased	[57]
Annexin VI isoform	Increased	[58]
Complement component 9	Increased	[58]
Ceruloplasmin	Increased	[55, 58]
Serum amyloid A4	Increased	[58]
Serum amyloid A2	Increased	[58]
Serum amyloid A1 isoform 2	Increased	[58]
Cystatin C	Increased	[59]
Neutrophil-activating peptide 2	Increased	[60]
Thrombin light chain	Increased	[61]
Growth-related oncogene alpha (GRO-alpha)	Increased	[61]
Alpha-1 acid glycoprotein	Increased	[62]
Haptoglobin	Increased	[63]
N terminus complement C3f	Decreased	[64]
Fibrinopeptide	Decreased	[64]
Complement C4 alpha peptides	Increased	[64]
Zyxin peptide	Increased	[64]
Coagulation factor XIII peptide	Increased	[64]
Biliverdin diglucuronide	Increased	[64]
Heat-shock protein 27	Increased	[65]
MYH2 protein	Increased	[66]
Mitochondrial ATP synthase	Increased	[66]
Sulphated glycoprotein-2	Increased	[66]
Glial fibrillary acidic protein	Increased	[66]

**Table 3:** Lung cancer serum biomarkers identified after MS-based studies.

Putative lung cancer biomarker	Expression in lung ca sera	References
Serum amyloid protein A	Increased	[71–76]
Haptoglobin alpha subunit	Increased	[77]
Hepatocyte growth factor	Increased	[77]
Transthyretin	Decreased	[75, 78]
Alpha-1 acid glycoprotein 1 and 2	Increased	[74]
Apolipoprotein A4 peptides	Increased/decreased	[79]
Fibrinogen alpha chain	Increased	[79]
Limbin	Increased	[79]

**Table 4:** Pancreatic cancer serum biomarkers identified after MS-based studies.

Putative pancreatic cancer biomarker	Expression in pancreatic ca sera	References
Apolipoprotein CIII	Increased	[82]
Serum amyloid protein A	Increased	[83]
Apolipoprotein A-II	Decreased	[84]
Apolipoprotein A-I	Decreased	[84]
Transthyretin	Decreased	[84]
Alpha-2 macroglobulin	Increased	[85]
Ceruloplasmin	Increased	[85]
Complement 3C	Increased	[85]
Platelet factor 4	Decreased	[86]
Mannose-binding lectin 2	Increased	[87]
Myosin light chain kinase 2	Increased	[87]
CXC chemokine ligand 7	Decreased	[88]
TIMP1-ICAM1	Increased	[89]
Alpha-1 antitrypsin	Increased	[90]
Fibrinogen gamma	Increased	[91]
C14orf166	Increased	[92]
Alpha-1 antichymotrypsin	Increased	[93]
